# Pathogen spectrum, clinical traits and exploration of mortality outcome-improving medication regimens in coinfection patients with severe fever with thrombocytopenia syndrome: a multicenter cohort study

**DOI:** 10.3389/fcimb.2025.1708979

**Published:** 2025-11-21

**Authors:** Zibo Fan, Lianhe Lu, Ling Lin, Jianping Duan, Qun Li, Chuanyun Zhao, Zhenghua Zhao, Yuanni Liu, Yong Zhou, Wei Zhou, Yi Shen, Zhihai Chen, Wei Zhang

**Affiliations:** 1 National Key Laboratory of Intelligent Tracking and Forecasting for Infectious Diseases, Beijing Ditan Hospital, Capital Medical University, Beijing, China; 2 Yantai Qishan Hospital, Yantai, Shandong, China; 3 Qingdao Infectious Disease Hospital, Qingdao, Shandong, China; 4 Taian City Central Hospital, Taian, Shandong, China; 5 Dalian Public Health Clinical Center, Dalian, Liaoning, China; 6 Dandong Infectious Disease Hospital, Dandong, Liaoning, China

**Keywords:** severe fever with thrombocytopenia syndrome, coinfection, bacteria, fungus, case fatality rate

## Abstract

**Purpose:**

Using a multicenter clinical data, this study aimed to systematically analyze the clinical characteristics, pathogen spectrum, and independent risk/protective factors for coinfection in SFTS patients; additionally, it sought to explore optimal therapeutic regimens to reduce the case fatality rate of SFTS patients with coinfection.

**Methods:**

Clinical data of 1,675 patients with SFTS who were hospitalized for the first time, collected from 6 institutions between May 2011 and November 2024, were included and analyzed.

**Results:**

The coinfection group had a significantly higher case fatality rate (24.3% vs. 6.7%, *P* < 0.001), with the mixed bacterial-fungal infection subgroup showing the highest mortality risk (Log-rank test, *P* < 0.001). Albumin served as a protective factor against coinfection, with a cutoff value of 32 g/L identified; administering human albumin at this threshold could partially reduce the coinfection rate. Furthermore, on the basis of this protective effect of albumin, the combination of human albumin and corticosteroids was associated with a trend toward improved prognostic survival in coinfected patients.

**Conclusion:**

This study found a high proportion of SFTS patients with coinfection, which significantly worsens their clinical prognosis. SFTS currently has no specific therapy; clinical treatment focuses on symptomatic and supportive care, with strict standardization for antibiotics and antifungal use, and attention to patients’ nutritional support. Albumin is a key protective factor associated with a lower risk of coinfection in SFTS patients; combined use of human albumin and corticosteroids was associated with a further trend toward better clinical prognosis in coinfected patients.

## 1 Introduction

Severe Fever with Thrombocytopenia Syndrome (SFTS) is an acute natural focal infectious disease caused by the Severe Fever with Thrombocytopenia Syndrome Virus (SFTSV). This virus belongs to the family *Phenuiviridae* of the order *Bunyavirales* (Peng et al., 2025; Xu et al., 2025). First reported in China in 2009, SFTS cases have kept increasing, with confirmed cases successively found in neighboring countries like South Korea and Japan, making it a globally concerned emerging infectious disease (Oh et al., 2014; Takahashi et al., 2014). Given its potential public health threat, World Health Organization (WHO) listed it as a priority emerging infectious disease for research in 2017.

As SFTS cases grow, a key clinical challenge stands out: the proportion of SFTS patients with bacterial or fungal coinfections has risen sharply, becoming critical to clinical prognosis. A study by scholars such as Zhang confirmed that the incidence of coinfection in SFTS patients ranges from 8% to 30%, which is a core factor leading to the deterioration of patients’ conditions and an increase in mortality, with fungal coinfections being particularly common (Zhang et al., 2022). The causes of this phenomenon are complex, mainly closely related to immune dysfunction, thrombocytopenia, and coagulation disorders induced by SFTSV infection itself. Moreover, the use of high-dose corticosteroids in clinical treatment further increases the risk of infection, making SFTS patients a high-risk group for coinfection (Li et al., 2018; Song et al., 2018).

Currently, research on coinfection in SFTS is limited. In a multicenter study by Yan Zuo et al., 443 SFTS patients were enrolled, and 190 of these patients with concurrent pulmonary infection were analyzed. The study identified age, time from onset to hospital admission, and skin manifestations as relevant risk factors (Zuo et al., 2023). Another study based on 6 years of clinical experience enrolled 269 SFTS patients and found that nearly half of them (43.87%, 118/269) were complicated with invasive pulmonary aspergillosis (IPA) (Wang et al., 2024). Few studies have explored mortality-improving medication regimens for SFTS patients with coinfection, and most suffer from limitations like small sample sizes and single-center designs.

This study focused on analyzing clinical characteristics and pathogen spectrum of SFTS patients with coinfection, identifying independent risk/protective factors for coinfection, and exploring regimens to lower their case fatality rate (CFR).

## 2 Materials and methods

### 2.1 Patients

**Figure 1:**
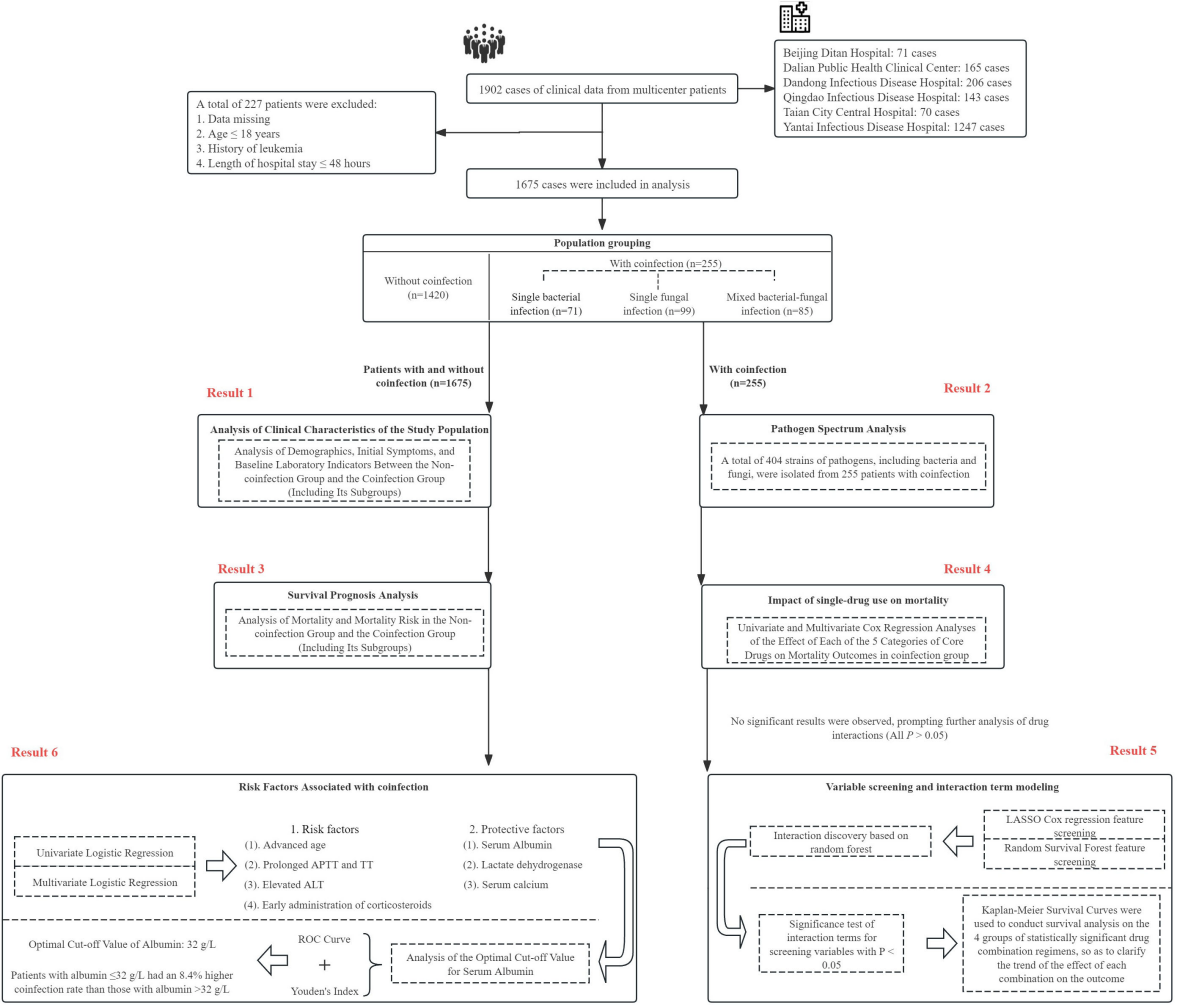
Flowchart of eligible patient screening and statistical analysis.

This multicenter retrospective study enrolled 1,902 SFTS patients who were hospitalized for the first time between May 2011 and November 2024. Among these patients, 1,675 met the inclusion criteria and were subsequently included in the final analysis (**Figure 1**). The centers involved in this study comprise 6 medical institutions: Beijing Ditan Hospital, Dalian Public Health Clinical Center, Dandong Infectious Disease Hospital, Qingdao Infectious Disease Hospital, Tai’an Central Hospital, and Yantai Infectious Disease Hospital. On admission, the clinical diagnosis of Severe Fever with Thrombocytopenia Syndrome (SFTS) was determined based on patients’ epidemiological information and clinical symptoms consistent with SFTS. Patients were laboratory-confirmed to have SFTS Virus (SFTSV) infection if real-time reverse transcript polymerase chain reaction (RT-PCR) detected SFTSV positively. Based on the study’s requirements, this study only included clinically diagnosed cases and confirmed cases for which a diagnosis had been made upon hospital admission.

The exclusion criteria are as follows: (1) Pathogen culture has been completed, but the data results are missing; (2) When *Corynebacrerium diphtheriae*, *Propionibacterium*, *Bacillus*, or *Micrococcus* are detected, these bacteria are directly identified as contaminating bacteria and thus not included in this study; (3) A history of hematological diseases such as leukemia and idiopathic thrombocytopenic purpura;(4) A history of autoimmune diseases; (5) Missing data accounting for more than 5% of the total medical record data. Without distinguishing between hospital-acquired and community-acquired coinfections, this study analyzed SFTS patients with coinfection from the comprehensive perspective of coinfection itself.

### 2.2 Study grouping and definitions

The non-coinfection group was defined as patients with no definite pathogen detected during hospitalization, and no infection-related symptoms, signs, or radiological evidence. The coinfection group included three subgroups: single bacterial infection, single fungal infection, and mixed bacterial-fungal infection. Single bacterial infection was defined as bacterial infection confirmed by the detection of definite pathogenic bacteria, accompanied by clinical symptoms and evidence of infection at the corresponding site, with exclusion of bacterial contamination or colonization; Single fungal infection was defined as confirmed by fungal detection in specimens, presence of fungal infection-related clinical manifestations and radiological features, positive fungus-specific tests (G test ≥ 100 pg/ml; galactomannan GM test via enzyme-linked immunosorbent assay ELISA, thresholds by specimen type: single serum/plasma ≥ 1.0 optical density index ODI, bronchoalveolar lavage fluid BALF ≥ 1.0 ODI, single serum/plasma ≥ 0.7 ODI plus BALF ≥ 0.8 ODI, or cerebrospinal fluid CSF ≥ 1.0 ODI) based on the European Organization for Research and Treatment of Cancer and the Mycoses Study Group (EORTC/MSG, 2019) (Donnelly et al., 2020).​ Mixed bacterial-fungal infection was defined as concurrent fulfillment of the diagnostic criteria for both bacterial infection and fungal infection during the course of SFTS.

In this study, medication records of six categories of core drugs, including corticosteroids, antibiotics, antifungals, human albumin, immunoglobulin, and immunopotentiators, were collected and analyzed. Detailed information regarding each drug category is provided in **Supplementary Table S1**. In this study, although some SFTS patients were administered either ribavirin or favipiravir, the two antiviral drugs, there is currently no clear evidence demonstrating their efficacy in treating SFTS patients. Therefore, these drugs were not included in the analysis of core therapeutic agents (Shen et al., 2024).

### 2.3 Data collection

Data on demographics, clinical signs, laboratory test results, microbial culture results, treatments, and outcomes were extracted from the electronic medical record system. The follow-up period concluded at patient discharge or death to determine the outcome (death or survival).

### 2.4 Statistical analysis

In this study, statistical analysis of data was performed using R software (Version 4.2) and SPSS software (Version 27.0), with the statistical significance level set at *P* < 0.05.

Non-normally distributed continuous data were presented as median (interquartile range) [M (IQR)], and the Mann-Whitney U test was used for inter-group comparison; categorical data were expressed as frequency (percentage) [n (%)], and the chi-square test (*χ*² test) was applied for inter-group comparison.

Kaplan-Meier (KM) curves were used to compare survival curves between the non-coinfection group and each coinfection subgroup, with the Log-rank test applied for intergroup comparisons. For 255 patients with coinfection, univariate and multivariate Cox proportional hazards regression models were used to assess the independent effect of individual drugs on mortality outcomes. To explore the impact of combined drug use on the survival outcomes of this group, a multi-drug interaction analysis strategy was adopted: LASSO Cox regression, Random Survival Forest (RSF), and interaction significance tests were used to screen pairwise drug combinations related to outcomes, and KM survival curves were used to visualize survival differences between groups. Additionally, univariate and multivariate logistic regression were employed to identify risk and protective factors for coinfection.

## 3 Results

### 3.1 Clinical characteristics of the study population

A total of 1,675 patients with SFTS were included in this study. Among them, there were 1,420 patients in the non-coinfection group and 255 patients in the coinfection group, with the latter consisting of three subgroups: 71 patients in the single bacterial infection group, 99 patients in the single fungal infection group, and 85 patients in the mixed bacterial-fungal infection group.

Compared with the non-coinfection group, all SFTS patients with coinfection (regardless of infection type) showed the following differences (**Supplementary Table S2**). Patients in the coinfection group were significantly older than those in the non-coinfection group (69 years vs. 64 years, *P* < 0.001). The prevalence rates of hypertension (24.3% vs. 17.6%, *P* = 0.014) and diabetes mellitus (13.7% vs. 8.1%, *P* = 0.006) in the coinfection group were significantly higher than those in the non-coinfection group. In the coinfection group, the incidences of fever (90.6% vs. 80.6%, *P* < 0.001), diarrhea (30.6% vs. 19.4%, *P* < 0.001), and arthralgia (27.5% vs. 19.7%, *P* = 0.007) were significantly higher, and fatigue was more common (85.5% vs. 77.7%, *P* = 0.005). In the coinfection group, the lymphocyte (lym) count decreased significantly (0.47 vs. 0.60×10^9^/L, *P* < 0.001), prothrombin time (PT), activated partial thromboplastin time (APTT), and thrombin time (TT) were all significantly prolonged (*P* < 0.001);Additionally, patients in the coinfection group generally presented with hypocalcemia (1.89 vs. 1.94 mmol/L), elevated renal function indicators (Urea. Ur and Creatinine. Cr), decreased albumin (ALB) (31.5 vs. 32.4 g/L), significantly reduced cholinesterase (CHE) levels (5483 vs. 5935.32 U/L), and significantly increased globulin (GLB) levels (26.2 vs. 25.41 g/L), with all *P* values < 0.001.

Based on the common characteristics of the coinfection group, when comparing its subgroups with the non-coinfection group, each subgroup exhibits unique differential features (**Table 1**). The single fungal infection group had the highest prevalence of hypertension (30.3% vs. 17.6%, *P* = 0.003);The Mixed bacterial-fungal infection group had the highest prevalence of diabetes mellitus (16.5% vs. 8.1%, *P* = 0.041);Among the clinical manifestations, the mixed bacterial-fungal infection group had the most prominent incidences of diarrhea (34.1% vs. 19.4%, *P* = 0.002), arthralgia (29.4% vs. 19.7%, *P* = 0.037), and nausea (28.2% vs. 40.4%, *P* = 0.030);Only in the fungal infection group was the globulin level significantly higher than that in the non-coinfection group (26.4 vs. 25.41 g/L, *P* < 0.001). The hemoglobin (Hb) level in the mixed bacterial-fungal infection group was significantly higher than that in the non-infection group (141 vs. 138 g/L, *P* = 0.040), and its CHE level showed the most significant decrease (5191 vs. 5935.32 U/L, P<0.001), while the decrease in the fungal infection group was relatively the mildest (5935 U/L vs. 5935.32 U/L, *P* = 0.018). At the same time, this group had the most significant renal function impairment, with Cr (77 vs. 66 μmol/L, *P* < 0.001) and Ur (6.88 vs. 5.41 mmol/L, *P* < 0.001) levels as indicators, and a lower platelet (PLT) count (52 vs. 60×10^9^/L, *P* = 0.012). All coinfection subgroups showed significantly prolonged PT, APTT, and TT, with the bacterial and fungal mixed infection group having the largest extension amplitude (all P<0.001).

**Table 1:** Comparison of demographic and clinical characteristics among different populations of hospitalized patients with SFTS.

Characteristics	Non-coinfection (*N* = 1420)	Bacterial infection (*N* = 71)	*P* ^a^	Fungal infection (*N* = 99)	*P* ^b^	Bacterial plus fungal infection (*N* = 85)	*P* ^c^
General information, median (IQR) or n (%)							
Age (years)	64 (55, 71)	70 (61, 74)	<0.001	68 (61, 74)	<0.001	69 (61, 74)	<0.001
Gender			0.144		0.146		0.911
Male	684 (48.2)	41 (57.7)		40 (40.4)		40 (47.1)	
Female	736 (51.8)	30 (42.3)		69 (69.7)		45 (52.9)	
History of underlying disease, n (%)							
Hypertension	250 (17.6)	15 (21.1)	0.429	30 (30.3)	0.003	17 (20)	0.560
Coronary Heart Disease	68 (4.8)	3 (4.2)	1.000	8 (8.1)	0.150	6 (7.1)	0.304
Diabetes	115 (8.1)	9 (12.7)	0.183	12 (12.1)	0.185	14 (16.5)	0.041
Cerebral infarction	51 (3.6)	2 (2.8)	1.000	4 (4.0)	0.779	3 (3.5)	1.000
Chronic hepatitis B	11 (0.8)	1 (1.4)	0.444	2 (2.0)	0.206	2 (2.4)	0.164
Intracerebral hemorrhage	10 (0.7)	3 (4.2)	0.021	1 (1.0)	0.525	1 (1.2)	0.474
Symptoms at admission, n (%)							
Fever	1145 (80.6)	68 (95.8)	<0.001	94 (94.9)	<0.001	78 (91.8)	0.009
Fatigue	1103 (77.7)	60 (84.5)	0.190	85 (85.9)	0.059	73 (85.9)	0.080
Lethargy	799 (56.3)	42 (59.2)	0.713	61 (61.6)	0.345	54 (63.5)	0.215
Palpitation	24 (1.7)	0	0.625	1 (1.0)	1.000	0	0.395
Muscle soreness	489 (34.4)	26 (36.6)	0.703	32 (32.3)	0.743	31 (36.5)	0.725
Arthralgia	280 (19.7)	17 (23.9)	0.364	28 (28.3)	0.052	25 (29.4)	0.037
Nausea	574 (40.4)	33 (46.5)	0.324	38 (38.4)	0.751	24 (28.2)	0.030
Diarrhea	276 (19.4)	19 (26.8)	0.129	30 (30.3)	0.013	29 (34.1)	0.002
Melena	19 (1.3)	1 (1.4)	1.000	1 (1.0)	1.000	1 (1.2)	1.000
Cough	208 (14.6)	14 (19.7)	0.234	19 (19.2)	0.242	8 (9.4)	0.166
Oliguria	59 (4.2)	1 (1.4)	0.361	0	0.029	1 (1.2)	0.253
State of consciousness			0.833		0.186		0.256
Awake	1240 (87.3)	0		85 (85.9)		72 (84.7)	
Somnolence	45 (3.2)	4 (5.6)		8 (8.1)		1 (1.2)	
Confusion	104 (7.3)	5 (7.0)		6 (6.1)		12 (14.1)	
Stupor	3 (0.2)	0		0		0	
Light coma	13 (0.9)	0		0		0	
Deep coma	5 (0.4)	0		0		0	
Delirium	10 (0.7)	0		0		0	
Cutaneous congestion	111 (7.8)	2 (2.8)	0.165	3 (3.0)	0.111	2 (2.4)	0.085
Rash	78 (5.5)	1 (1.4)	0.175	1 (1.0)	0.057	1 (1.2)	0.126
Bulbar conjunctival edema	110 (7.7)	2 (2.8)	0.164	3 (3.0)	0.110	2 (2.4)	0.085
Lymphadenopathy	246 (17.3)	9 (12.7)	0.418	20 (20.2)	0.494	18 (21.2)	0.378
Hemorrhage	30 (2.1)	1 (1.4)	1.000	1 (1.0)	0.717	2 (2.4)	0.701
Bilateral renal percussion tenderness	49 (3.5)	1 (1.4)	0.512	0	0.071	0	0.109
Laboratory variables, median (IQR)							
White blood cell (10^9/L)	2.35 (1.53, 3.90)	2.43 (1.49, 4.35)	0.735	2.14 (1.45, 3.66)	0.284	2.35 (1.53, 3.70)	0.789
Neutrophil (10^9/L)	1.33 (0.83, 2.32)	1.68 (0.94, 3.39)	0.037	1.34 (0.89, 2.29)	0.895	1.36 (0.94, 2.60)	0.204
Lymphocyte (10^9/L)	0.60 (0.38, 1.04)	0.43 (0.31, 0.73)	<.0.001	0.47 (0.32, 0.74)	0.004	0.46 (0.32, 0.71)	<0.001
Monocyte (10^9/L)	0.16 (0.08, 0.38)	0.12 (0.06, 0.30)	0.098	0.18 (0.08, 0.49)	0.337	0.13 (0.07, 0.47)	0.521
Eosinophil (10^9/L)	0 (0, 0)	0 (0, 0)	0.953	0 (0, 0)	0.148	0 (0, 0)	0.959
Red blood cell (10^12/L)	4.52 (4.20, 4.89)	4.41 (4, 4.88)	0.322	4.53 (4.18, 4.93)	0.630	4.57 (4.29, 5.07)	0.138
Hemoglobin (g/L)	138 (127, 148)	141 (128, 154)	0.161	138 (128, 152)	0.317	141 (128, 156)	0.040
Mean corpuscular volume (fl)	88.8 (85.72, 91.90)	90 (86.8, 92.7)	0.099	88.2 (85.9, 91.7)	0.999	89.8 (85.6, 93.5)	0.085
Mean corpuscular hemoglobin concentration (g/L)	342 (333, 350)	345 (334, 353)	0.242	342 (333, 350)	0.633	340 (330, 354.5)	0.827
Red cell distribution width-coefficient of variation (%)	12.8 (12.2, 13.6)	12.6 (12.1, 13.7)	0.506	12.7 (12.2, 13.3)	0.144	12.7 (12.1, 13.5)	0.109
Platelet count (10^9/L)	60 (44, 82)	52 (38, 69)	0.005	60 (48, 74)	0.661	52 (40, 70.5)	0.012
Mean platelet volume (fl)	10.7 (10, 11.48)	10.7 (9.8, 11.2)	0.363	10.6 (9.9, 11.4)	0.372	10.6 (9.9, 11.5)	0.697
Lactate dehydrogenase (U/L)	609.5 (384.25, 900)	649 (437, 900)	0.168	676 (424, 900)	0.479	609.5 (465.5, 900)	0.480
Creatine kinase (U/L)	376.6 (167, 841.13)	488 (243, 1009)	0.036	501 (273, 1044)	0.001	393 (317, 121.5)	0.004
Creatine Kinase-MB (U/L)	17 (11.69, 29)	15 (9, 29)	0.109	16 (10, 22)	0.036	15 (8, 24.5)	0.018
Serum potassium (mmol·L)	3.8 (3.5, 41)	3.83 (3.5, 4.12)	0.305	3.8 (3.4, 4.1)	0.969	3.8 (3.5, 4.1)	0.739
Serum sodium (mmol·L)	135 (132, 138)	134.5 (131.5, 137)	0.405	135 (132, 137)	0.877	135 (134, 137.7)	0.100
Serum chlorine (mmol·L)	99 (96, 102)	99 (95.3, 101)	0.136	99 (95, 101)	0.186	99 (96, 103.15)	0.546
Serum calcium (mmol·L)	1.94 (1.87, 2.03)	1.88 (1.8, 1.95)	<0.001	1.9 (1.77, 1.96)	<0.001	1.88 (1.77, 1.96)	<0.001
Urea (mmol/L)	5.41 (3.98, 7.08)	6.71 (5, 8.21)	0.001	5.82 (4.31, 9.29)	0.011	6.88 (5.41, 9.89)	<0.001
Creatinine(μmol/L)	66 (54, 79.18)	68 (58.1, 82)	0.227	67 (57, 81)	0.158	77 (64.5, 95.2)	<0.001
Prothrombin time (s)	12.4 (11.6, 12.9)	12.9 (12.4, 13.4)	<0.001	12.7 (12.1, 13.4)	<0.001	13.2 (12.5, 14.1)	<0.001
Activated partial thromboplastin time (s)	43.6 (38.3, 47.2)	47.9 (43.6, 57.3)	<0.001	46 (43.6, 53.1)	<0.001	48.8 (43.6, 58.4)	<0.001
Thrombin time (s)	20 (17.8, 21.3)	21.9 (19.1, 27.6)	<0.001	21.9 (20, 25.7)	<0.001	22.7 (20, 31.6)	<0.001
Alanine amiotransferase (U/L)	75 (47.53, 112.8)	75 (50.9, 152.3)	0.306	75 (57, 111)	0.225	75 (74.9, 127.3)	0.023
Aspartate aminotransferase (U/L)	118.95 (68, 224.38)	125.6 (62.2, 294)	0.285	118.95 (68, 207)	0.782	119 (57.85, 248.6)	0.488
Total bilirubin (μmol·L)	9.66 (7.56, 12.8)	9.53 (8.09, 11.21)	0.459	9.66 (8.04, 12.26)	0.475	9.66 (8.42, 12.66)	0.347
Total protein (g/L)	57.8 (54.2, 61.6)	57.3 (53.9, 59.4)	0.186	58.2 (55.5, 63.6)	0.030	57.8 (53.3, 61.2)	0.150
Albumin (g/L)	32.4 (30, 35.3)	31.72 (28.69, 33.4)	0.004	31.6 (28.7, 34)	0.006	31.3 (28.1, 33.05)	<0.001
Globulin (g/L)	25.41 (22.7, 21.75)	26.4 (23.08, 28.8)	0.77	26.4 (25, 29.7)	<0.001	25.5 (23.55, 28.7)	0.072
Gamma-Glutamyl Transferase (U/L)	33 (22, 60)	32 (22, 54)	0.302	33 (21, 57)	0.523	33 (20.5, 63.5)	0.799
Alkaline Phosphatase (U/L)	63 (52.85, 77.95)	63 (48.7, 80.35381)	0.828	63 (49.6, 91.9)	0.939	63 (51.2, 73.7)	0.513
Cholinesterase (U/L)	5935.32 (5291.75, 6881)	5510 (4517, 6276.24)	<0.001	5935 (4704, 6528.71)	0.018	5191 (4339, 5935)	<0.001

SFTS, severe fever with thrombocytopenia syndrome.

*P*^a^, Comparison between the Bacterial infection Group and the Non-coinfection Group.

*P*^b^, Comparison between the Fungal infection Group and the Non-coinfection Group.

*P*^c^, Comparison between the Bacterial plus fungal infection Group and the Non-coinfection Group.

### 3.2 Pathogen spectrum and clinical specimen distribution characteristics of SFTS patients with coinfections

A total of 404 pathogens were detected in this study, covering three major categories: Gram-negative bacteria (GNB), Gram-positive bacteria (GPB), and fungi, and their distribution in different specimen types showed significant specificity (**Table 2**). Fungi were the most predominant type of pathogens (49.01%, 198/404), among which *Aspergillus (Asp.)* was the dominant fungus, accounting for 56.57% (112/198) of the total fungi. GNB were the second most common (40.35%, 163/404), mainly including *Klebsiella pneumoniae* (*Kpn*) (25.15%, 41/163), *Acinetobacter baumannii* (*Ab*) (24.54%, 40/163), and *Escherichia coli* (*E. coli*) (19.02%, 31/163). Finally, GPB accounted for (10.6%, 43/404), mainly including *Staphylococcus epidermidis* (*S. epi*) (20.93%, 9/43) and *Staphylococcus aureus* (*S. aureus*) (27.91%, 12/43).

**Table 2:** Distribution of pathogen isolates across specimen types.

Pathogen	Blood [N (%)]	Feces [N (%)]	Sputum [N (%)]	Urine [N (%)]	Marrow [N (%)]	Oral Swab [N (%)]	Total [N (%)]
Gram-negative bacteria							
*Acinetobacter baumannii*	5 (9.62)	0	35 (12.37)	0	0	0	40 (9.9)
*Escherichia coli*	9 (17.31)	0	12 (4.24)	10 (62.5)	0	0	31 (7.67)
*Pseudomonas aeruginosa*	8 (15.38)	0	17 (6.01)	0	0	0	25 (6.19)
*Klebsiella oxytoca*	0	0	1 (0.35)	0	0	0	1 (0.25)
*Klebsiella pneumoniae*	0	0	40 (14.13)	1 (6.25)	0	0	41 (10.15)
*Streptococcus pneumoniae*	0	0	2 (0.71)	0	0	0	2 (0.5)
*Haemophilus influenzae*	0	0	7 (2.47)	0	0	0	7 (1.73)
*Morganella*	0	0	1 (0.35)	0	0	0	1 (0.25)
*Stenotrophomonas maltophilia*	0	0	2 (0.71)	0	0	0	2 (0.5)
*Burkholderia cepacia*	0	0	4 (1.41)	0	0	0	4 (0.99)
*Enterobacter cloacae*	0	0	1 (0.35)	0	0	0	1 (0.25)
*Moraxella catarrhalis*	0	0	6 (2.12)	0	00	0	6 (1.49)
*Acinetobacter haemolyticus*	0	0	0	2 (12.5)	0	0	2 (0.5)
Gram-positive bacteria							
*Staphylococcus epidermidis*	6 (11.54)	0	3 (1.06)	0	0	0	9 (2.23)
*Enterococcus faecalis*	2 (3.85)	0	0	2 (12.5)	0	0	4 (0.99)
*Streptococcus gordonii*	1 (1.92)	0	0	0	0	0	1 (0.25)
*Streptococcus mitis*	3 (5.77)	0	1 (0.35)	0	0	0	4 (0.99)
*Staphylococcus hominis*	4 (7.69)	0	1 (0.35)	0	1 (50)	0	6 (1.49)
*Staphylococcus haemolyticus*	1 (1.92)	0	1 (0.35)	0	0	0	2 (0.5)
*Enterococcus faecium*	2 (3.85)	0	0	0	0	0	2 (0.5)
*Staphylococcus aureus*	3 (5.77)	0	9 (3.18)	0	0	0	12 (2.97)
*Oral Streptococci*	3 (5.77)	0	0	0	0	0	3 (0.74)
Fungus							
*Aspergillus*	2 (3.85)	0	109 (38.52)	0	1 (50)	0	112 (27.72)
*Unspecified fungus*	0	0	18 (6.36)	1 (6.25)	0	39 (78)	58 (14.36)
*Candida albicans*	3 (5.77)	1 (100)	13 (4.59)	0	0	11 (22)	28 (6.93)
Total	52 (100)	1 (100)	283 (100)	16 (100)	2 (100)	50 (100)	404 (100)

This table shows the types and composition ratios of pathogens isolated from various specimens of SFTS patients with coinfection.

Sputum samples were the main source of pathogens (70.05%, 283/404). Among these pathogens, fungi accounted for the highest proportion (49.47%, 140/283), and *Asp*. was particularly dominant among the fungi (77.86%, 109/140). The second most common were GNB (45.23%, 128/283), mainly including *Kpn* (31.25%, 40/128) and *Ab* (27.34%, 35/128). A total of 52 pathogen strains were detected in blood specimens, among which GPB were dominant (48.08%, 25/52). Among the GPB, the main pathogenic strains included *S. epi* (24%, 6/25), *Staphylococcus hominis* (*S.hom*) (16%, 4/25), as well as *Streptococcus mitis* (*S. mit*), *S. aureus*, and *Streptococcus oralis* (*S. ora*); each of these three strains accounted for 12% (3/25). In addition, a small number of *Candida albicans* (*C. alb*) and *Asp* were also detected in the fungi category. The pathogenic bacteria in urine specimens were monomorphic, with a total of 16 strains detected. Among these strains, GNB were the most prevalent (81.25%, 13/16), and this group was predominantly composed of *E. coli* (76.92%, 10/13). A total of 50 pathogenic strains were detected from oral swabs, all of which were fungi. These fungi consisted of unclassified fungi (78%, 39/50) and *C. alb* (22%, 11/50).

### 3.3 Survival and prognosis analysis

Comparison between the coinfection group and the non-coinfected group (**Supplementary Table S3**): The CFR was 6.7% (95/1420) in the non-coinfection group, while the overall CFR of the coinfection group reached 24.3% (62/255), with a statistically significant difference between the two groups (*P* < 0.001). Comparison of each coinfection subgroup vs. non-coinfection group (**Table 3**): Among subgroups, the bacterial-fungal mixed infection group had the highest CFR (30.5%, 30/85). All three subgroups had significantly higher CFR than the non-co-infection group (all *P ≤* 0.013).

**Table 3:** Clinical outcomes and therapeutic medications in hospitalized patients with SFTS.

Characteristics	Non-coinfection (*N* = 1420)	Bacterial infection (*N* = 71)	*P* ^a^	Fungal infection (*N* = 99)	*P* ^b^	Bacterial plus fungal infection (*N* = 85)	*P^c^*
LOS	10 (6, 13)	9 (4, 13)	0.137	10 (8, 15)	0.038	12 (5.5, 17.5)	0.019
Death	95 (6.7)	18 (25.4)	<0.001	14 (14.1)	0.013	30 (30.5)	<0.001
Antifungal agents	20 (1.4)	4 (5.6)	0.024	47 (47.5)	<0.001	43 (50.6)	<0.001
Immunopotentiators	120 (8.5)	0	0.005	3 (3.0)	0.056	3 (3.5)	0.150
Corticosteroids	247 (17.4)	31 (43.7)	<0.001	52 (52.5)	<0.001	52 (61.2)	<0.001
Antibiotics	654 (46.1)	40 (56.3)	0.112	65 (65.7)	<0.001	59 (69.4)	<0.001
Gamma globulin	115 (8.1)	4 (5.6)	0.652	14 (14.1)	0.059	3 (3.5)	0.148
Human albumin	123 (8.7)	10 (14.1)	0.132	21 (21)	<0.001	5 (5.9)	0.546

LOS, Length of stay; SFTS, severe fever with thrombocytopenia syndrome.

*P*^a^, Comparison between the Bacterial infection Group and the Non-coinfection Group.

*P*^b^, Comparison between the Fungal infection Group and the Non-coinfection Group.

*P*^c^, Comparison between the Bacterial plus fungal infection Group and the Non-coinfection Group.

**Figure 2:**
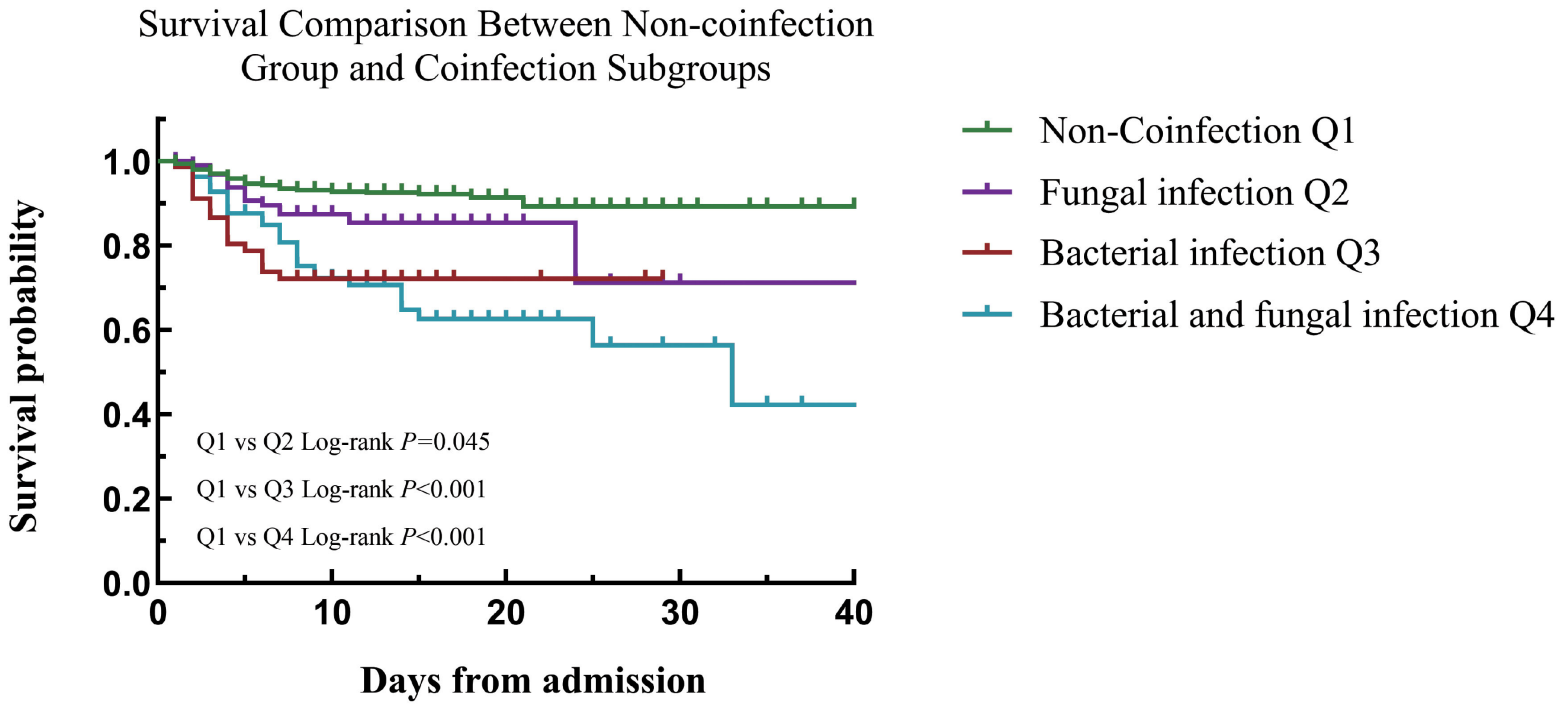
Kaplan - Meier survival curves: non - coinfection vs. coinfection subgroups.

Focusing on mortality risk: KM analysis was used to estimate survival, and log-rank test showed significant differences in mortality risk among groups with the non-coinfection group as the reference. The bacterial-fungal mixed infection group had the highest mortality risk (*P* < 0.001), followed by the single bacterial infection group (*P* < 0.001) and the single fungal infection group (*P* = 0.045) (**Figure 2**).

### 3.4 Analysis of the correlation between medication strategy and mortality outcome

#### 3.4.1 Correlation between medication frequency and infection status: intergroup validation based on survival differences

Since the above analysis has confirmed a statistically significant difference in survival outcomes between the non-coinfection and coinfection groups, we hypothesize that, besides infection-related complications and organ damage, medication strategy may be a potential regulatory factor. To validate this hypothesis, we compared the use frequency of core therapeutic drugs across groups; results showed significant stratified characteristics in medication distribution among groups (**Table 3**). Focusing on antifungal use frequency (vs. non-co-infection group): the single bacterial infection group was slightly higher (5.6%, *P* = 0.024); the single fungal infection group (47.5%, *P* < 0.001) and bacterial-fungal mixed infection group (50.6%, *P* < 0.001) were significantly higher. Antibiotic use frequency (vs. non-coinfection group): fungal infection group (65.7%, P<0.001) and bacterial-fungal mixed infection group (69.4%, *P* < 0.001) showed significantly higher frequencies. Corticosteroids use: the non-coinfection group had the lowest rate (17.4%); all coinfection subgroups showed significantly higher rates (*P* < 0.001), with an increasing trend by infection complexity: single bacterial infection group (43.7%) < single fungal infection group (52.5%) < bacterial-fungal mixed group (61.2%). Human albumin use: only the fungal infection group had a significantly higher frequency (21%, *P* < 0.001) vs. the non-co-infection group (8.7%).

#### 3.4.2 Correlation between monotherapy use and mortality outcome in the total coinfection group

**Figure 3:**
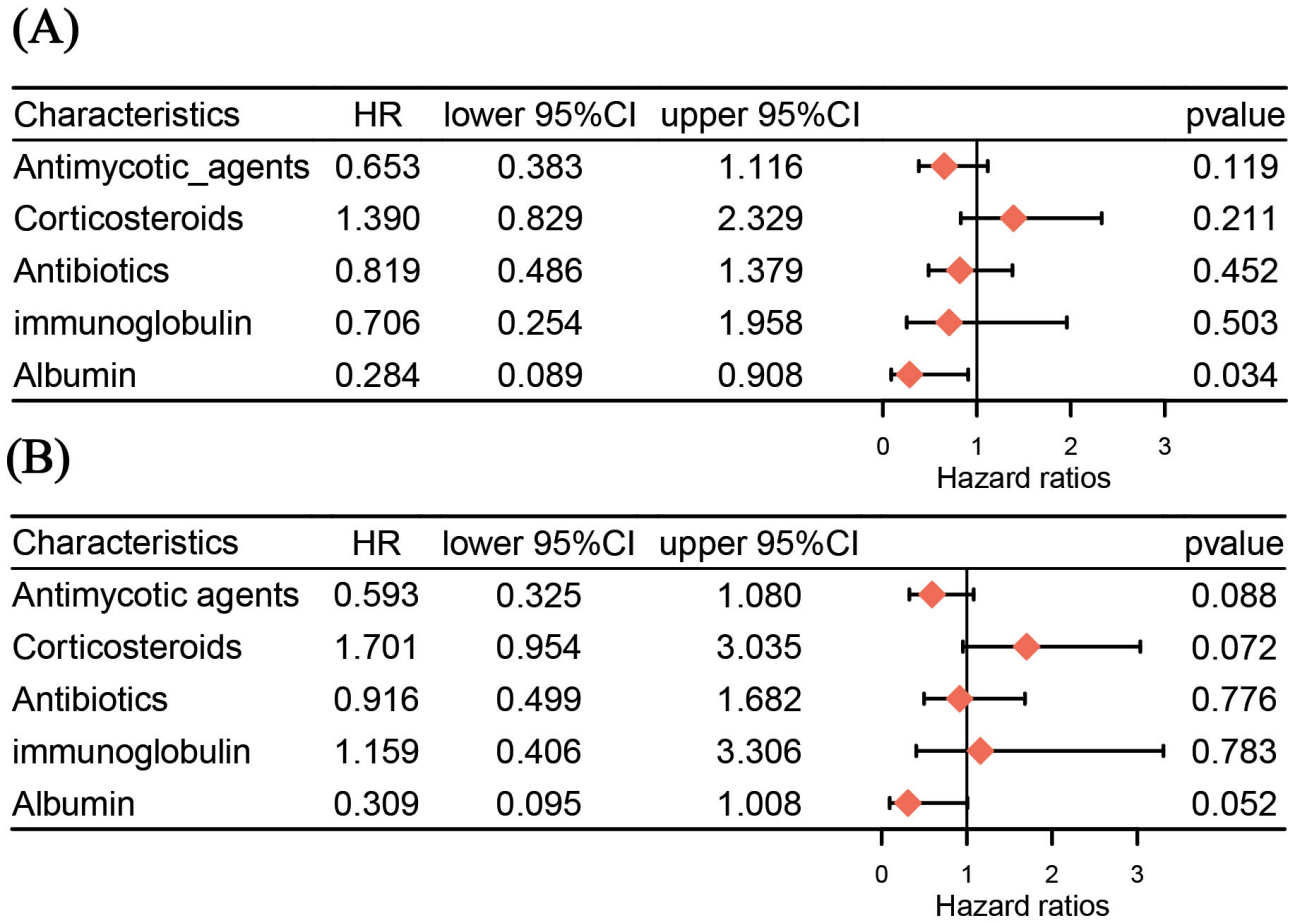
Cox regression forest plot: analyzing the impact of different therapeutic drugs on survival outcomes. **(A)** Univariate Cox regression forest plot (no confounding adjustment); **(B)** Multivariate Cox regression forest plot (with confounding adjustment). The adjusted hazard ratio [HR, shown in panel **(B)**] was derived from a Cox regression model, which accounted for the influences of factors such as sex, age, underlying diseases, and clinical medications. CI, confidence interval; HR, hazard ratio.

Medication frequency differences only reflect macro distribution, failing to clarify monotherapy’s effect direction or independent impact on outcome risk. Thus, we focused on the coinfection group (n=225) and used Cox proportional hazards regression to analyze monotherapy use vs. mortality outcome, identifying real clinical effects of various drugs (**Figure 3**). Immunopotentiators were excluded from the statistical analysis due to the presence of extreme values. After adjusting for confounders (age, gender, underlying disease, and other medications), multivariate Cox regression showed that none of the 5 core drugs (antibiotics, antifungals, Corticosteroids, human albumin, immunoglobulin) had a statistically significant effect on mortality outcome (all *P*>0.05), though some exhibited potential risk-modulating trends.

#### 3.4.3 Correlation between polypharmacy use and mortality outcome in the coinfection group

Since the above analysis showed no significant monotherapy effect, we propose that the synergistic or antagonistic effects of polypharmacy may be the more critical driver of outcome differences in SFTS patients with coinfection. We first modeled two-drug combinations in the coinfection group using a two-step approach: LASSO survival analysis for **Supplementary Figures S1A, B**, plus Random Survival Forest (RSF) for **Supplementary Figure S1C** to initially identify potentially relevant combinations.

The top 10 strongest drug interaction combinations identified by RSF analysis are shown in **Supplementary Figure S1C**. Of the 10 drug combinations, 4 showed statistical significance in interaction tests (*P* < 0.05) as follows: albumin and corticosteroids (Depth=0.521783, *P* = 0.005); albumin and immunoglobulin (Depth=0.546267, *P* = 0.02); albumin and antibiotics (Depth=0.549183, *P* = 0.02); corticosteroids and immunoglobulin (Depth=0.533367, *P* = 0.045). Details in **Supplementary Table S4**.

**Figure 4:**
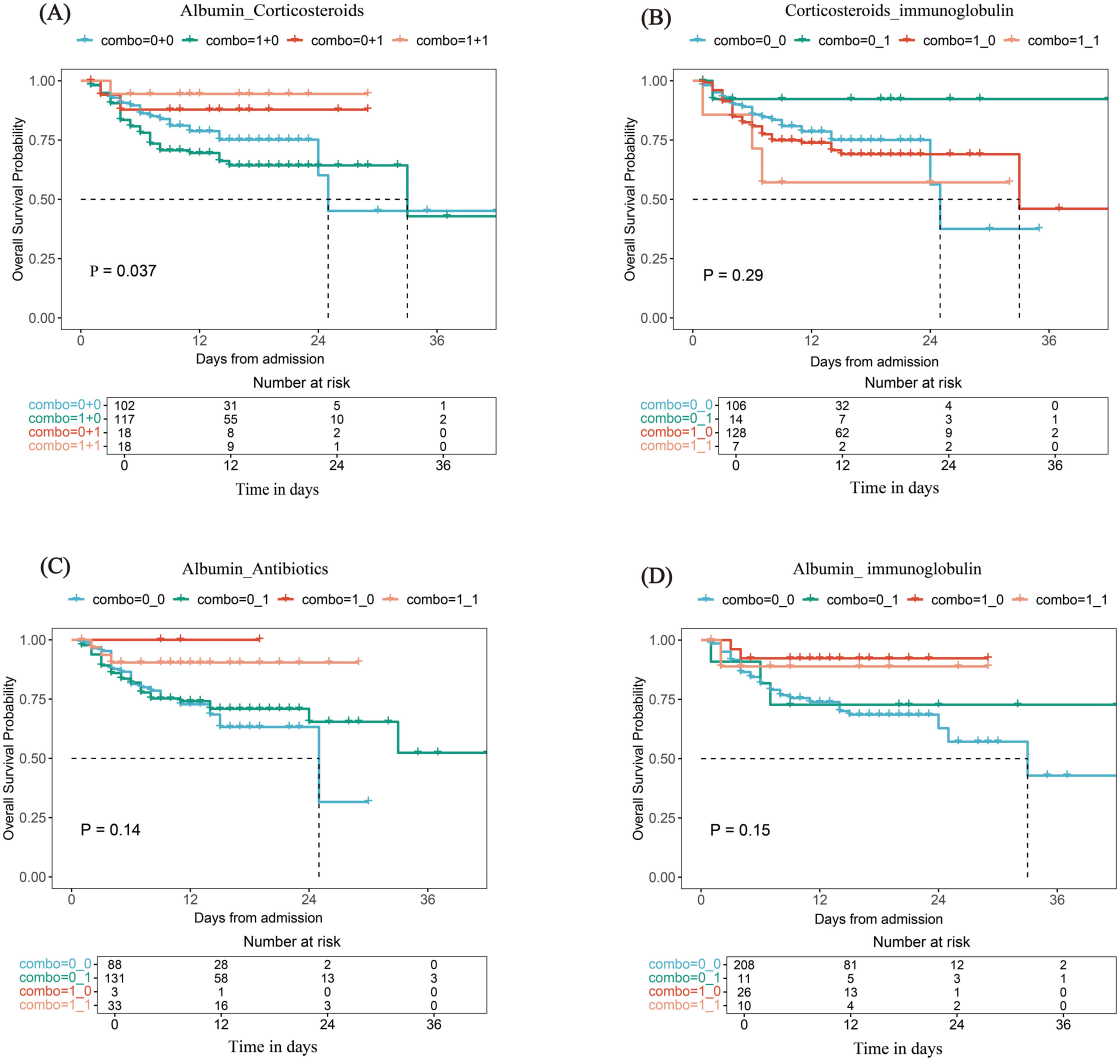
Kaplan - Meier survival curves: impact of different drug combinations on survival outcomes. **(A)** Albumin and Corticosteroids, **(B)** Corticosteroids and Immunoglobulin, **(C)** Albumin and Antibiotics, **(D)** Albumin and Immunoglobulin.

The 4 combinations’ impact on survival outcomes was further analyzed via KM curves (**Figure 4**). Only albumin and corticosteroids showed significant survival differences (*P* = 0.037), initially indicating a protective trend (**Figure 4A**); the rest had no statistical significance (**Figures 4B-D**).

### 3.5 Risk/protective factors associated with coinfection in SFTS patients

Owing to the significant role of coinfection in adverse outcomes, we utilized logistic regression analysis to analyze the risk and protective factors related to the occurrence of coinfection (**Table 4**). Based on univariate results, multivariate analysis was performed to exclude confounder effects. Following multivariate adjustment, it was revealed that advanced age, prolonged APTT, prolonged TT, elevated ALT, and corticosteroid use within 48 hours of admission were independent risk factors for coinfection in SFTS patients; in contrast, CHE, ALB, and Ca served as independent protective factors for coinfection in these patients.

**Table 4:** Risk/protective factors associated with coinfection in SFTS patients.

Characteristics	Univariate analysis		Multivariate analysis	
	OR (95%CI)	*P* values	OR (95%CI)	*P* values
Age (years)	1.039 (1.026-1.052)	<0.001	1.026 (1.011-1.042)	<0.001
Lymphocyte (10^9/L)	0.484 (0.359-0.654)	<0.001		
Hemoglobin (g/L)	1.002 (1.000-1.004)	0.095		
Platelet count (10^9/L)	0.995 (0.991-0.999)	0.016		
Lactate dehydrogenase (U/L)	1.000 (0.999-1.000)	0.011	0.999 (0.998-0.999)	<0.001
Creatine Kinase-MB (U/L)	1.000 (1.000-1.000)	0.060		
Serum calcium (mmolL)	0.069 (0.030-0.160)	<0.001	0.255 (0.093-0.698)	0.008
Urea (mmol/L)	1.021 (1.003-1.039)	0.022		
Creatinine(μmol/L)	1.002 (1.000-1.004)	0.048		
Prothrombin time (s)	1.035 (0.995-1.075)	0.086		
Activated partial thromboplastin time (s)	1.025 (1.017-1.033)	<0.001	1.012 (1.001-1.023)	0.036
Thrombin time (s)	1.055 (1.039-1.071)	<0.001	1.026 (1.009-1.044)	0.003
Alanine amiotransferase (U/L)	1.001 (1.000-1.002)	0.005	1.002 (1.001-1.004)	<0.001
Albumin (g/L)	0.925 (0.897-0.954)	<0.001	0.942 (0.903-0.983)	0.006
Cholinesterase (U/L)	1.000 (1.000-1.000)	<0.001		
Corticosteroids use within the first 48 h	0.187 (0.141-0.248)	<0.001	4.710 (3.430-6.467)	<0.001

The table presents results of univariate and multivariate logistic regression analyses. OR (95% CI) = odds ratio (95% confidence interval); Variables with statistically significant associations in multivariate analysis (P < 0.05) are retained as independent associated factors for coinfection in SFTS patients.

SFTS, severe fever with thrombocytopenia syndrome.

**Figure 5:**
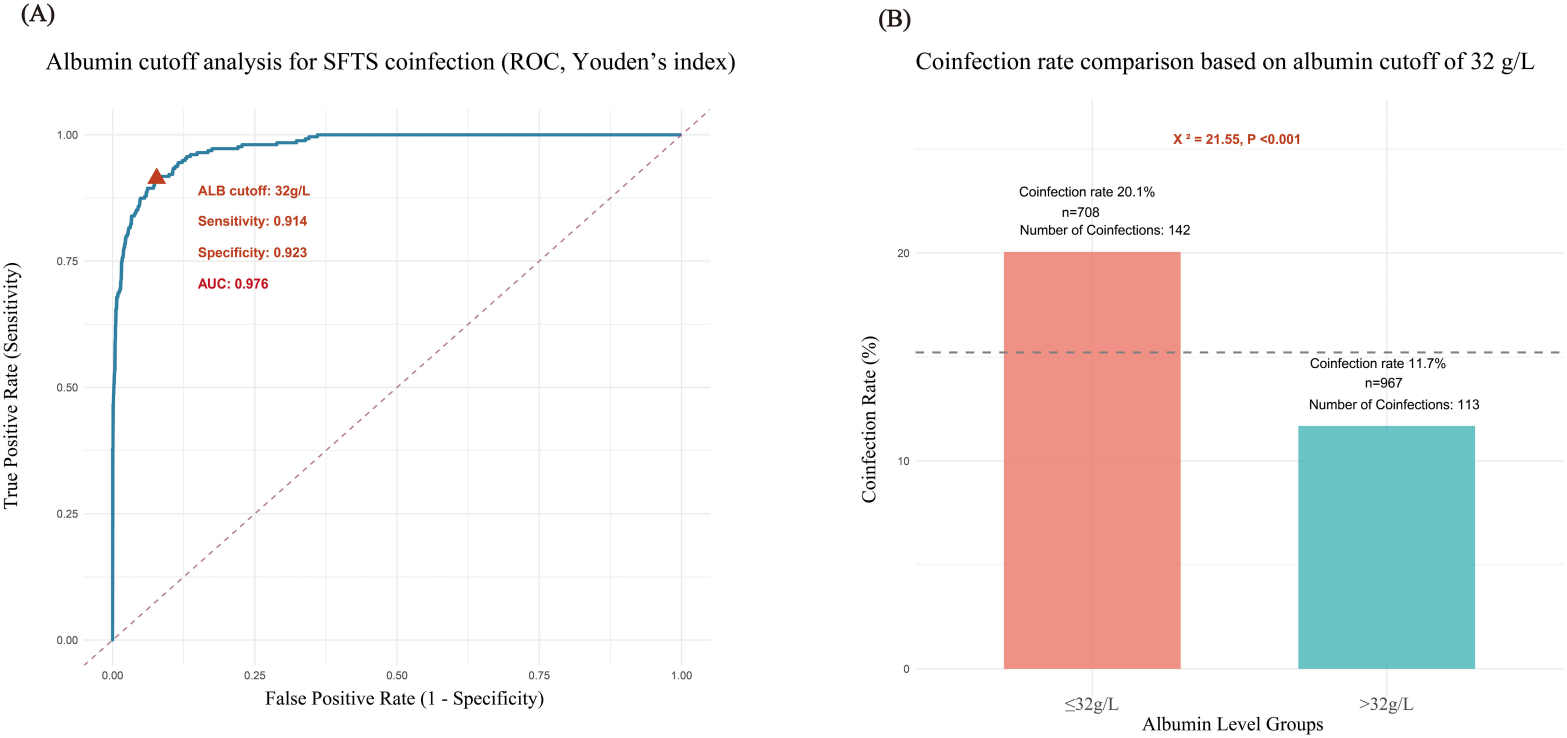
Cutoff value analysis of serum albumin in patients with coinfection and non – coinfection. **(A)** ROC Analysis Combined with Youden’s Index for Determining Serum Albumin Cutoff Value, **(B)** Differences in Coinfection Rates Based on Serum Albumin Distribution with 32 g/L as the Baseline.

Given albumin’s role as both a protective factor against coinfection and an effective therapeutic agent, we further explored its clinical intervention threshold. Among all 1675 patients, ROC curves for albumin levels were plotted using coinfection as the outcome variable (**Figure 5A**). Results showed an AUC of 0.976 for ALB levels in predicting coinfection. The Youden index was calculated, and 32 g/L albumin was finally determined as the optimal intervention cutoff. A bar chart comparing coinfection rates based on the 32 g/L albumin threshold (**Figure 5B**) intuitively showed that the coinfection rate was 20.1% in the albumin ≤32 g/L group, while it significantly decreased to 11.7% in the albumin >32 g/L group. The difference between the two groups was statistically significant (χ²=21.55, *P* < 0.001).

## 4 Discussion

This study detected 404 pathogens. The lungs were the most susceptible site, and sputum the primary specimen for pathogen detection: fungi dominated sputum (49.47%, 140/283), with *Asp* accounting for 77.86% (109/140). GNB were the next most common (45.23%, 128/283), dominated by *Kpn* (31.25%, 40/128) and *Ab* (27.34%, 35/128); these two are the main species causing secondary bacterial infections in SFTS patients. The second most common specimen for pathogen detection was blood, followed by urine, and this is roughly consistent with Hong Han Ge et al.’s study (Ge et al., 2022). Additionally, bacterial and fungal mixed infection accounted for 33.33% of the coinfection group (85/255), and this proportion has not been fully addressed in previous studies. Among SFTS patients undergoing oral swab specimen detection, the rate of fungal detection was 100% (50/50). In conjunction with the clinical observation that most patients with pulmonary fungal infections had pre-existing oral fungal infections, this implies a possible link between the two. In recent years, with the increase in the number of cases of SFTS co-infections, co-infections involving spotted fever group rickettsiae (SFGR) and Orientia tsutsugamushi cannot be ignored either. Previous studies have shown that co-infection with spotted fever group rickettsiae leads to delayed recovery and an increased risk of death in patients (Lu et al., 2016; Zhang et al., 2019). A South Korean patient was co-infected with SFTSV and two genotypes (Boryong and Taguchi) of Orientia tsutsugamushi. Given their similar clinical manifestations but the higher mortality rate of SFTS, enhanced clinical differential diagnosis is required to avoid misdiagnosis (Yoo et al., 2018).

From the perspective of medication data, the use of antibiotics and antifungals has clear targeting. However, Cox regression analysis showed that neither drug had a significant protective effect on mortality outcomes. This does not mean that antibiotics and antifungals are ineffective. Based on the characteristics of the pathogen spectrum, antibiotics can precisely cover dominant bacteria, while antifungals can specifically target major fungi. Their role is merely limited to eradicating secondary pathogens (Hotchkiss et al., 2016). The core pathological damage of SFTS is the cellular immune suppression and immune paralysis mediated by the SFTSV itself, which in turn leads to the excessive production of proinflammatory and anti-inflammatory mediators (Sun et al., 2012; Suzuki et al., 2020; Bryden et al., 2022). Thus, antibiotics and antifungals cannot significantly alter the trajectory of adverse outcomes jointly driven by the virus and infection. The adverse outcomes of SFTS are jointly driven by the combined effects of the virus’s core damage and secondary infections, rather than by a single infectious factor. Additionally, the use of glucocorticoids carries significant risks. Cox regression analysis showed a slightly increased risk trend for mortality associated with glucocorticoid use (HR = 1.39, *P* = 0.072).

However, the human albumin plus corticosteroids combination identified in this study provides a new approach to balance the risks of corticosteroids and overcome therapeutic challenges. Its advantage lies in not being limited to pathogen-targeted treatment, but focusing instead on addressing the pathological processes of SFTS. Mechanistically, albumin is first and foremost a key regulatory factor in infection prognosis. Albumin maintains vascular endothelial integrity, preserves acid-base balance, protects the microcirculation and tissue damage associated with inflammatory processes, and simultaneously mitigates the immunosuppressive risks of corticosteroids. This provides a stable tissue environment for corticosteroids to exert their anti-inflammatory effects, preventing multiorgan damage caused by the spread of inflammatory mediators (Ferrer et al., 2018; Fajgenbaum and June, 2020). This ensures their anti-inflammatory activity while reducing side effects caused by excessively high free concentrations (Abeydeera et al., 2018). Interestingly, this combination also complements antibiotics and antifungals. Albumin optimizes pharmacokinetics: for antibacterial agents with high protein binding rates such as β-lactams (including ceftriaxone, ertapenem) and aminoglycosides, normal serum albumin levels maintain proper drug-protein binding ratios, providing a favorable pharmacokinetic environment for antibacterial agents to act stably in the body (Contreras et al., 1994; Garot et al., 2011). Notably, albumin also exerts a synergistic effect with certain antifungal agents. A study by Petros Ioannou et al. demonstrated that albumin supplementation increased the binding of caspofungin to Aspergillus hyphae by 5-fold. This suggests that albumin may act as a potential carrier molecule to enhance the efficacy of antifungal agents (Ioannou et al., 2015).

The early identification and prediction of coinfection in SFTS patients remain a major challenge. Through multivariate analysis, we screened out a series of baseline indicators, which can serve as important parameters for the early prediction of coinfection. These indicators are consistent with the pathological mechanisms and clinical characteristics of SFTS, as well as the conclusions of previous studies, thus providing a reference basis for the early identification, prevention, and control of coinfection in SFTS patients (Abeydeera et al., 2018; Ge et al., 2022). Notably, in addition to albumin, indicators such as CHE and Ca are also independent protective factors against coinfection. This further suggests that clinical practice should attach importance to the maintenance of patients’ nutritional status.

For SFTS patients with coinfection, if they present with hypoalbuminemia and marked inflammatory responses, early combination therapy of corticosteroids and human albumin may be considered on the basis of targeted anti-infective treatment. Albumin is not only a core indicator for nutritional support but also participates in immune regulation. This aligns with our team’s previous research (Zhang et al., 2025).

Our study has limitations. First, SFTS patients with coinfection, especially coinfection subgroups had small sample sizes. To avoid reduced statistical power or bias, single drug effect and drug interaction analyses were based on the overall infection group, with no subgroup-stratified analyses. Thus, the conclusion that corticosteroids combined with albumin improves survival is mainly from total coinfection group data and needs cautious interpretation; its subgroup-specific effects require verification by larger-sample studies. Future prospective studies with larger samples are needed to optimize regimens and validate risk prediction model extrapolability.

## 5 Conclusion

This study found a high proportion of SFTS patients with coinfection, which significantly worsens their clinical prognosis. SFTS currently has no specific therapy; clinical treatment focuses on symptomatic and supportive care, with strict standardization for antibiotics and antifungal use, and attention to patients’ nutritional support. Albumin is a key protective factor associated with a lower risk of coinfection in SFTS patients; combined use of human albumin and corticosteroids was associated with a further trend toward better clinical prognosis in coinfected patients.

## Data Availability

The raw data supporting the conclusions of this article will be made available by the authors, without undue reservation.

## Supplementary material

Supplementary Figure 1 Analysis of drug interactions based on LASSO regression and random survival forest (RSF). **(A)** LASSO Cox Regression Coefficient Path Plot for Drug Variable Selection, **(B)** LASSO Cox Regression Cross-Validation Plot for Drug Variable Selection, **(C)** Random Survival Forest (RSF) Analysis Ranking Plot of Drug Interaction Combinations in Patients with Coinfections.
