# Supplementary material for: Pathogen spectrum, clinical traits and exploration of mortality outcome-improving medication regimens in coinfection patients with severe fever with thrombocytopenia syndrome: a multicenter cohort study
Source: Front Cell Infect Microbiol. 2025 Nov 21;15:1708979. doi: 10.3389/fcimb.2025.1708979 (PMC12678257; doi:10.3389/fcimb.2025.1708979)
Supplement: Supplementary Figure 1 — Analysis of drug interactions based on LASSO regression and random survival forest (RSF). (A) LASSO Cox Regression Coefficient Path Plot for Drug Variable Selection, (B) LASSO Cox Regression Cross-Validation Plot for Drug Variable Selection, (C) Random Survival Forest (RSF) Analysis Ranking Plot of Drug Interaction Combinations in Patients with Coinfections. [file Table1.docx]

Supplementary Material

# **Supplementary Tables**

**Supplementary Table 1.** Medication for SFTS Patients with Coinfection versus Those without Coinfection.

| Medication | Non-coinfection  (*N*=1420) | Bacterial infection  (*N*=71) | *P* ^a^ | Fungal infection  (*N*=99) | *P* ^b^ | Bacterial plus fungal infection (*N*=85) | *P ^c^* |
| --- | --- | --- | --- | --- | --- | --- | --- |
| **Antifungal agents** | 20 (1.4) | 4 (5.6) | 0.024 | 47 (47.5) | <0.001 | 43 (50.6) | <0.001 |
| Fluconazole | 17 (1.2) | 0 | 1.000 | 26 (26.3) | <0.001 | 11 (12.9) | <0.001 |
| Voriconazole | 3 (0.2) | 4 (5.6) | <0.001 | 24 (24.2) | <0.001 | 36 (42.4) | <0.001 |
| **Antibiotics** | 654 (46.1) | 40 (56.3) | 0.112 | 65 (65.7) | <0.001 | 59 (69.4) | <0.001 |
| Broad-Spectrum Cephalosporin | 235 (16.5) | 11 (15.5) | 1.000 | 18 (18.2) | 0.676 | 11 (12.9) | 0.452 |
| Piperacillin sodium sulbactam sodium | 96 (6.7) | 24 (33.8) | <0.001 | 34 (34.3) | <0.001 | 36 (42.4) | <0.001 |
| Aminoglycosides | 9 (0.6) | 0 | 1.000 | 1 (1.0) | 0.491 | 0 | 1.000 |
| Carbapenem | 48 (3.4) | 4 (5.6) | 0.308 | 14 (14.1) | <0.001 | 24 (28.2) | <0.001 |
| Tetracyclines | 235 (16.5) | 7 (9.9) | 0.185 | 8 (8.1) | 0.023 | 9 (10.6) | 0.173 |
| Fluoroquinolones | 250 (17.6) | 19 (26.8) | 0.058 | 30 (30.3) | 0.003 | 31 (36.5) | <0.001 |
| Penicillin | 49 (3.5) | 1 (1.4) | 0.512 | 2 (2.0) | 0.770 | 3 (3.5) | 1.000 |
| Vancomycin | 4 (0.3) | 0 | 1000 | 0 | 1.000 | 0 | 1.000 |
| Macrolide | 34 (2.4) | 1 (1.4) | 1.000 | 1 (1.0) | 0.724 | 1 (1.2) | 0.78 |
| **Immunopotentiators** | 120 (8.5) | 0 | 0.005 | 3 (3.0) | 0.056 | 3 (3.5) | 0.150 |
| Thymopentin | 84 (5.9) | 0 | 0.030 | 1 (1.0) | 0.039 | 1 (1.2) | 0.085 |
| Thymosin α1 | 31 (2.2) | 0 | 0.397 | 0 | 0.260 | 0 | 0.415 |
| Interleukin-11 | 6 (0.4) | 0 | 1.000 | 2 (2.0) | 0.091 | 2 (2.4) | 0.071 |
| **Corticosteroids** | 247 (17.4) | 31 (43.7) | <0.001 | 52 (52.5) | <0.001 | 52 (61.2) | <0.001 |
| Methylprednisolone | 79 (5.6) | 14 (19.7) | <0.001 | 30 (30.3) | <0.001 | 38 (44.7) | <0.001 |
| Dexamethasone | 112 (7.9) | 4 (5.6) | 0.651 | 5 (5.1) | 0.433 | 10 (11.8) | 0.216 |
| Hydrocortisone | 72 (5.1) | 15 (21.1) | <0.001 | 23 (23.2) | <0.001 | 14 (16.5) | <0.001 |
| **Gamma globulin** | 115 (8.1) | 4 (5.6) | 0.652 | 14 (14.1) | 0.059 | 3 (3.5) | 0.148 |
| **Human albumin** | 123 (8.7) | 10 (14.1) | 0.132 | 21 (21) | <0.001 | 5 (5.9) | 0.546 |

Abbreviations: SFTS, severe fever with thrombocytopenia syndrome.

*P*^a^: Comparison between the Bacterial infection Group and the Non-coinfection Group.
*P*^b^: Comparison between the Fungal infection Group and the Non-coinfection Group.
*P*^c^: Comparison between the Bacterial plus fungal infection Group and the Non-coinfection Group.

**Supplementary Table 2.** Comparison of Clinical Characteristics Between SFTS Hospitalized Patients with and Without Coinfection.

| Characteristics | Non-coinfection  (*N*=1420) | Coinfection  (*N*=255) | *P* |
| --- | --- | --- | --- |
| **General information, median (IQR) or n (%)** |  |  |  |
| Age (years) | 64 (55, 71) | 69 (61, 74) | <0.001 |
| Gender |  |  | 0.839 |
| Male | 684 (48.2) | 121 (47.5) |  |
| Female | 736 (51.8) | 1345 (52.5) |  |
| **History of underlying disease, n (%)** |  |  |  |
| Hypertension | 250 (17.6) | 62 (24.3) | 0.014 |
| Coronary Heart Disease | 68 (4.8) | 17 (6.7) | 0.215 |
| Diabetes | 115 (8.1) | 35 (13.7) | 0.006 |
| Cerebral infarction | 51 (3.6) | 9 (3.5) | 1.000 |
| Chronic hepatitis B | 11 (0.8) | 5 (2.0) | 0.082 |
| Intracerebral hemorrhage | 10 (0.7) | 5 (2.0) | 0.064 |
| **Symptoms at admission, n (%)** |  |  |  |
| Fever | 1145 (80.6) | 231 (90.6) | <0.001 |
| Fatigue | 1103 (77.7) | 218 (85.5) | 0.005 |
| Lethargy | 799 (56.3) | 157 (61.6) | 0.131 |
| Palpitation | 24 (1.7) | 1 (0.4) | 0.159 |
| Muscle soreness | 489 (34.4) | 89 (34.9) | 0.886 |
| Arthralgia | 280 (19.7) | 70 (27.5) | 0.007 |
| Nausea | 574 (40.4) | 95 (37.3) | 0.367 |
| Diarrhea | 276 (19.4) | 78 (30.6) | <0.001 |
| Melena | 19 (1.3) | 3 (1.2) | 1.000 |
| Cough | 208 (14.6) | 41 (16.1) | 0.556 |
| Oliguria | 59 (4.2) | 2 (0.8) | 0.005 |
| State of consciousness |  |  | 0.187 |
| Awake | 1240 (87.3) | 219 (85.9) |  |
| Somnolence | 45 (3.2) | 13 (5.1) |  |
| Confusion | 104 (7.3) | 23 (9.0) |  |
| Stupor | 3 (0.2) | 0 |  |
| Light coma | 13 (0.9) | 0 |  |
| Deep coma | 5 (0.4) | 0 |  |
| Delirium | 10 (0.7) | 0 |  |
| Cutaneous congestion | 111 (7.8) | 7 (2.7) | 0.002 |
| Rash | 78 (5.5) | 3 (1.2) | 0.001 |
| Bulbar conjunctival edema | 110 (7.7) | 7 (2.7) | 0.003 |
| Lymphadenopathy | 246 (17.3) | 47 (18.4) | 0.655 |
| Hemorrhage | 30 (2.1) | 4 (1.6) | 0.809 |
| Bilateral renal percussion tenderness | 49 (3.5) | 1 (0.4) | 0.004 |
| **Laboratory variables, median (IQR)** |  |  |  |
| White blood cell (10^9/L) | 2.35 (1.53, 3.90) | 2.3 (1.51, 3.71) | 0.539 |
| Neutrophil (10^9/L) | 1.33 (0.83, 2.32) | 1.41 (0.91, 2.6) | 0.070 |
| Lymphocyte (10^9/L) | 0.60 (0.38, 1.04) | 0.47 (0.32, 0.71) | <0.001 |
| Monocyte (10^9/L) | 0.16 (0.08, 0.38) | 0.15 (0.07, 0.39) | 0.545 |
| Eosinophil (10^9/L) | 0 (0, 0) | 0 (0, 0.01) | 0.391 |
| Red blood cell (10^12/L) | 4.52 (4.20, 4.89) | 4.54 (4.18, 4.96) | 0.546 |
| Hemoglobin (g/L) | 138 (127, 148) | 140 (128, 154) | 0.016 |
| Mean corpusular volume (fl) | 88.8 (85.72, 91.90) | 89 (86.3, 92.6) | 0.078 |
| Mean corpusular hemoglobin concerntration (g/L) | 342 (333, 350) | 342 (332, 351) | 0.456 |
| Red cell distribution width-coefficient of variation (%) | 12.8 (12.2, 13.6) | 12.7 (12.1, 13.4) | 0.032 |
| Platelet count (10^9/L) | 60 (44, 82) | 56 (43, 72) | 0.002 |
| Mean platelet volume (fl) | 10.7 (10, 11.48) | 10.6 (9.9, 11.3) | 0.232 |
| Lactate dehydrogenase (U/L) | 609.5 (384.25, 900) | 636 (441, 900) | 0.135 |
| Creatine kinase (U/L) | 376.6 (167, 841.13) | 475 (285, 1044) | <0.001 |
| Creatine Kinase-MB (U/L) | 17 (11.69, 29) | 15 (9.05, 24) | <0.001 |
| Serum potassium (mmol·L) | 3.8 (3.5, 4.1) | 3.8 (3.5, 4.1) | 0.503 |
| Serum sodium (mmol·L) | 135 (132, 138) | 135 (132, 137) | 0.695 |
| Serum chlorine (mmol·L) | 99 (96, 102) | 99 (95.4, 101.4) | 0.231 |
| Serum calcium (mmol·L) | 1.94 (1.87, 2.03) | 1.89 (1.78, 1.95) | <0.001 |
| Urea (mmol/L) | 5.41 (3.98, 7.08) | 6.3 (4.96, 9.03) | <0.001 |
| Creatinine(μmol/L) | 66 (54, 79.18) | 70 (60, 85) | <0.001 |
| Prothrombin time (s) | 12.4 (11.6, 12.9) | 12.9 (12.4, 13.5) | <0.001 |
| Activeated partial thromboplasting time (s) | 43.6 (38.3, 47.2) | 47.8 (43.6, 55.9) | <0.001 |
| Thrombin time (s) | 20 (17.8, 21.3) | 22.1 (19.9, 27.6) | <0.001 |
| Alanine amiotransferase (U/L) | 75 (47.53, 112.8) | 75 (57.2, 129) | 0.013 |
| Aspartate aminotransferase (U/L) | 118.95 (68, 224.38) | 118.95 (64, 237.7) | 0.963 |
| Total bilirubin (μmol·L) | 9.66 (7.56, 12.8) | 9.66 (8.15, 12.13) | 0.569 |
| Total protein (g/L) | 57.8 (54.2, 61.6) | 57.8 (54.3, 61.5) | 0.875 |
| Albumin (g/L) | 32.4 (30, 35.3) | 31.5 (28.6, 33.4) | <0.001 |
| Globulin (g/L) | 25.41 (22.7, 21.75) | 26.2 (24.3, 29.3) | <0.001 |
| Gamma-Glutamyl Transferase (U/L) | 33 (22, 60) | 33 (21, 56) | 0.451 |
| Alkaline Phosphatase (U/L) | 63 (52.85, 77.95) | 63 (50.1, 80.3) | 0.609 |
| Cholinesterase (U/L) | 5935.32 (5291.75, 6881) | 5483 (4532, 6274) | <0.001 |

**Abbreviation:** SFTS, severe fever with thrombocytopenia syndrome.

**Supplementary Table 3.** Clinical Outcomes and Therapeutic Medications Between SFTS Hospitalized Patients with and Without Coinfection.

| Characteristics | Non-coinfection  (*N*=1420) | Coinfection  (*N*=255) | *P* |
| --- | --- | --- | --- |
| LOS | 10 (6, 13) | 10 (6, 15) | 0.077 |
| Death | 95 (6.7) | 62 (24.3) | <0.001 |
| Antifungal agents | 20 (1.4) | 94 (36.9) | <0.001 |
| Immunopotentiators | 120 (8.5) | 6 (2.4) | <0.001 |
| Corticosteroids | 247 (17.4) | 135 (52.9) | <0.001 |
| Antibiotics | 654 (46.1) | 164 (64.3) | <0.001 |
| Gamma globulin | 115 (8.1) | 21 (8.2) | 0.901 |
| Human albumin | 123 (8.7) | 36 (14.1) | 0.010 |

**Abbreviation:** LOS, Length of stay.

**Supplementary Table 4.** Analysis results of medication combination interactions in coinfection group (Random Survival Forest Model).

| Medication 1 | Medication 2 | Depth | *P* |
| --- | --- | --- | --- |
| Albumin | Corticosteroids | 0.521783 | 0.005 |
| Albumin | Immunoglobulin | 0.546267 | 0.02 |
| Albumin | Antibiotics | 0.549183 | 0.02 |
| Albumin | Antimycotic_agents | 0.620467 | 0.051 |
| Corticosteroids | Immunoglobulin | 0.533367 | 0.045 |
| Antibiotics | Antimycotic_agents | 0.759717 | 0.55 |
| Antimycotic_agents | Immunoglobulin | 0.641833 | 0.845 |
| Antibiotics | Immunoglobulin | 0.741133 | 0.895 |
| Antimycotic_agents | Corticosteroids | 0.668533 | 0.94 |
| Antibiotics | Corticosteroids | 0.752333 | 0.96 |

Medication 1 and Medication 2 refer to the two medications in each medication combination. Depth reflects interaction intensity.

# **Supplementary Figures**

**Supplementary Figure 1. Analysis of Drug Interactions Based on LASSO Regression and Random Survival Forest (RSF).**





**Supplementary Figure 1.** Analysis of Drug Interactions Based on LASSO Regression and Random Survival Forest (RSF).

(A) LASSO Cox Regression Coefficient Path Plot for Drug Variable Selection, (B) LASSO Cox Regression Cross-Validation Plot for Drug Variable Selection, (C) Random Survival Forest (RSF) Analysis Ranking Plot of Drug Interaction Combinations in Patients with Coinfections.
